# Non-mycosis fungoides cutaneous lymphomas in a referral center in Taiwan: A retrospective case series and literature review

**DOI:** 10.1371/journal.pone.0228046

**Published:** 2020-01-24

**Authors:** Kwei-Lan Liu, Wen-Chien Tsai, Chih-Hung Lee

**Affiliations:** 1 Department of Dermatology, Kaohsiung Chang Gung Memorial Hospital and Chang Gung University College of Medicine, Kaohsiung, Taiwan; 2 Huang PH Dermatology and Aesthetics, Kaohsiung, Taiwan; Universita degli Studi di Roma La Sapienza, ITALY

## Abstract

**Background:**

While mycosis fungoides (MF) and Sézary syndrome (SS) are the most common cutaneous lymphomas (CLs), there is limited data about non-MF/SS CLs.

**Objective:**

We aimed to evaluate clinical characteristics of non-MF/SS CLs.

**Methods:**

A retrospective analysis evaluated patients with non-MF/SS CLs covering a period of 17 years. The records of 59 patients with non-MF/SS CLs were reviewed for demographic profiles, clinical features, and survival outcomes.

**Results:**

Our series consisted of 38 non-MF/SS cutaneous T-cell lymphomas (CTCLs) and 21 cutaneous B-cell lymphomas (CBCLs). In the group of non-MF/SS CTCLs including 33 primary and five secondary cases, there were cases of anaplastic large cell lymphoma (15.3% of non-MF/SS CLs), extranodal natural killer/ T-cell lymphoma (13.5%), peripheral T-cell lymphoma, not otherwise specified (13,5%), adult T-cell leukemia/lymphoma (8.5%), subcutaneous panniculitis-like T-cell lymphoma (6.8%) and angioimmunoblastic T-cell lymphoma (6.8%). In the group of CBCLs including nine primary and 12 secondary cases, there were cases of diffuse large B-cell lymphoma (22.0%), mantle cell lymphoma (5.1%), extranodal marginal lymphoma of mucosa associated lymphoid tissue (3.4%), follicle center lymphoma (3.4%) and intravascular large B-cell lymphoma (1.7%). The overall survivals were 57 months for non-MF/SS CTCLs and 41.5 months for CBCLs. Elevated serum lactate dehydrogenase level, thrombocytopenia, multiple anatomical sites of skin involvement and lower albumin level may be associated with poor prognosis in non-MF/SS CTCLs, but the latter two were not in CBCLs.

**Conclusion:**

With this series, we hope to provide indigenous data and outcome of non-MF/SS CLs. The overall survival of non-MF/SS CTCLs was better than CBCLs.

## Introduction

Cutaneous lymphomas (CLs) are the second most common group of extranodal lymphomas [1, 2]. The incidence of CLs has increased in the past decades [3, 4]. There are primary and secondary CLs, which contain many different entities. These entities have heterogeneous clinical, histological and prognostic features. Primary CLs are defined as non-Hodgkin lymphomas (NHLs) arising in the skin without evidence of extracutaneous involvement at the time of diagnosis [2]. Primary CLs represent a rare heterogeneous entity of mature T-cell and natural killer (NK) cell neoplasms (CTCLs) and mature B-cell neoplasms (CBCLs), with mycosis fungoides (MF) comprising the majority of cases [5]. The classification of primary CLs has changed over the last few decades [1–3, 5, 6]. Secondary CLs are cutaneous infiltrates of disseminated nodal or extranodal lymphomas with various disease progression and survival outcomes [2].

According to the World Health Organization (WHO)-European Organization of Research and Treatment of Cancer (EORTC) classification for CLs [2, 3], primary CTCLs represent about 80% of primary CLs and consist of MF, primary cutaneous CD30+ lymphoproliferative disorders, subcutaneous panniculitis-like T-cell lymphoma, primary cutaneous CD4+ small/medium lymphoproliferative disorder, Sézary syndrome (SS), extranodal NK/T-cell lymphoma, nasal type (ENKL), adult T-cell leukemia/lymphoma (ATLL), primary γ/δ T-cell lymphoma, primary CD8+ aggressive epidermotropic cytotoxic T-cell lymphoma, and primary cutaneous peripheral T-cell lymphoma, not otherwise specified (pcPTCL-NOS). Primary CBCLs consist of extranodal marginal zone lymphoma of mucosa-associated lymphoid tissue (MALT lymphoma), primary cutaneous follicle center lymphoma and primary cutaneous diffuse large B-cell lymphoma.

Epidemiological studies in Korea showed geographical differences of CLs between Korea and Western countries [7]. Clinicopathologic analysis of CLs revealed a higher frequency of ENKL and a lower frequency of MF in Taiwan than in Western population [8]. MF is characterized by an indolent clinical course, a long-term evolution, and typical manifestations in the patch-or-plaque stage, with variable progression to the tumor stage and erythroderma. However, the prognosis and survival of non-MF/SS CLs are relatively miserable. There were one case series of MF [9], and two case series of CLs including MF and other entities reported in Taiwan [8, 10]. Few studies focusing on non-MF/SS CLs were conducted in Taiwan. We retrospectively collected non-MF/SS CLs patients in a referral center in southern Taiwan and investigate epidemiologic, clinical and laboratory features and survival outcomes indigenously.

## Materials and methods

Approval for this retrospective chart review was obtained from the Institutional Review Board of Chang Gung Memorial Hospital, Kaohsiung Medical Center, in Kaohsiung City, Taiwan. Because this was a retrospective study without revealing any personal data, the Institutional Review Board waived the requirement to obtain informed consent from all subjects including from a parent and/or legal guardian of subjects under age 18. All methods were carried out in accordance with relevant guidelines and regulations of the Institutional Review Board.

From January 1998 to December 2014, patients with diagnosis of CLs were identified using the pathology database of the Department of Dermatology of the Chang Gung Memorial Hospital, Kaohsiung Medical Center. Patients were excluded if the medical record was not available for the clinical diagnosis and laboratory data due to the remote past. This query generated cases with both clinically and pathologically confirmed CLs.

The demographic profiles, clinical presentation, lab examination, and survival outcomes of all the patients were collected. Primary CLs were defined as lymphomas presenting in the skin without evidence of extracutaneous involvement except regional lymph node at the time of diagnosis [2]. Conversely, the diagnosis of secondary CLs was established if there is evidence of extracutaneous involvement except regional lymph node at the time of diagnosis [2]. Age at diagnosis was determined using the date the pathologic diagnosis was made. Elevated serum LDH level was defined as greater than 240 units per liter, the upper limit of the normal range in the referral center. Thrombocytopenia was defined as lower than 150,000 per microliter. The lower limit of normal albumin range was 35 grams per liter in the referral center.

The final diagnosis of CLs was made according to the 2008 WHO classification of Hematopoietic and lymphoid Tumors and its 2016 update, and after correlating the clinical and pathologic findings [6]. The staging of CLs was based on the new tumor-node-metastasis-blood classification proposed by the International Society for Cutaneous Lymphomas and the CL force of the EORTC [11]. All statistical analyses were performed using SPSS (version 17; Chicago, IL, USA).

## Results

### Demographic profiles

In our data set, there were 26 patients with MF and 59 patients with non-MF/SS CLs. These patients were all Taiwanese. The 59 non-MF/SS CLs patients consisted of 38 CTCLs and 21 CBCLs. Thirty-three primary CTCLs, five secondary CTCLs, nine primary CBCLs and 12 secondary CBCLs were identified. Among the 59 non-MF/SS CLs patients listed in Table 1 (details in S1 Table), the male to female ratio was approximately 1:1 with a small male preponderance (male:female = 32:27). The 38 cases of non-MF/SS CTCLs included 21 males and 17 females. The 21 cases of CBCLs included 11 males and 10 males. It was distinctly unique that all four patients with subcutaneous panniculitis-like T-cell lymphoma were females without a past history of lupus erythematosus. However, all three patients with mantle cell lymphoma were males.

**Table 1:** Basic demographic characteristics of the patients.

Classification	Number of cases (N)	Frequency (%)	Gender, M:F		Age at diagnosis (years)	
			Ratio	N	Median	Range
**Primary cutaneous T-cell lymphoma**	33	55.9	1.2	18:15	51	12–80
**Extranodal natural killer/T-cell lymphoma**	8	13.5	1.67	5:3	58	24–80
**Peripheral T-cell lymphoma, not otherwise specified**	8	13.5	3	6:2	57	37–75
**Primary cutaneous anaplastic large-cell lymphoma**	8	13.5	1.67	5:3	31	12–69
**Adult T-cell leukemia/lymphoma**	5	8.5	0.67	2:3	66	61–74
**Subcutaneous panniculitis-like T-cell lymphoma**	4	6.8	0	0:4	44	35–58
**Secondary cutaneous T-cell lymphoma**	5	8.5	1.5	3:2	48	26–71
**Angioimmunoblastic T-cell lymphoma**	4	6.8	3	3:1	56	46–71
**Systemic anaplastic T-cell lymphoma**	1	1.7	0	0:1	26	-
**Primary cutaneous B-cell lymphoma**	9	15.3	0.8	4:5	76	47–86
**Primary cutaneous diffuse large B-cell lymphoma**	6	10.2	1	3:3	76	47–86
**Extranodal marginal lymphoma of mucosa associated lymphoid tissue (MALT lymphoma)** ^a^	1	1.7	-	1:0	52	-
**Primary cutaneous follicle center lymphoma**	1	1.7	0	0:1	76	-
**Intravascular large B-cell lymphoma**	1	1.7	0	0:1	71	-
**Secondary cutaneous B-cell lymphoma**	12	20.3	1.4	7:5	65.5	35–71
**Diffuse large B cell lymphoma**	7	11.8	0.75	3:4	66	35–70
**Mantle cell lymphoma**	3	5.1	-	3:0	71	60–71
**MALT lymphoma** ^a^	1	1.7	-	1:0	60	-
**Follicle center lymphoma**	1	1.7	0	0:1	65	-

^a^ MALT lymphoma in the 2008 World Health Organization (WHO) classification corresponds to marginal zone lymphoma in the 2005 WHO-European Organization for Research and Treatment of Cancer classification.

The average ages of non-MF/SS CTCLs and CBCLs were 51 and 65 years old, respectively. The median age of CBCLs patients (median 71, range 35–86 years) was significantly older than that of the non-MF/SS CTCLs group (median 55, range 12–80 years). The patients with primary CBCLs (median 76, range 47–86 years) were also significantly older than the patients with non-MF/SS primary CTCLs (median 51, range 12–80 years). The age of patients with anaplastic large-cell lymphoma was notably young (median 31, range 12–69 years).

### Relative frequencies

The 42 non-MF/SS primary CLs patients consisted of 33 cases of CTCLs and nine cases CBCLs and the 17 secondary CLs patients included five cases of and 12 cases of CBCLs (Table 1). The relatively common non-MF/SS primary CTCLs were ENKL (eight cases, 13.5% of non-MF/SS CLs), pcPTCL-NOS (eight cases, 13.5%) and anaplastic large-cell lymphoma (eight cases, 13.5%). ATLL (five cases, 8.5%) and subcutaneous panniculitis-like T-cell lymphoma (four cases, 6.8%) were also common. The most frequently found secondary CTCL was angioimmunoblastic T cell lymphoma (four cases, 6.8%).

In the group of primary CBCLs, primary cutaneous diffuse large B-cell lymphoma was most common (six cases, 10.2% of non-MF/SS CLs). MALT lymphoma (one case, 1.7%), primary cutaneous follicle center lymphoma (one case, 1.7%) and intravascular large B-cell lymphoma (one case, 1.7%) were also seen. Diffuse large B cell lymphoma (seven cases, 11.8%) was also the most common cause of secondary CBCLs, followed by mantle cell lymphoma (three cases, 5.1%), MALT lymphoma (one case, 1.7%) and follicle center lymphoma (one case, 1.7%).

### Clinical characteristics and staging

The anatomical sites of skin involvement in descending order of frequency were limbs (28 cases, 47.4% of non-MF/SS CLs), trunk (23 cases, 39.0%) and followed by head and neck (19 cases, 32.2%). Both non-MF/SS primary CTCLs and primary CBCLs involved limbs most commonly.

**Fig 1:**
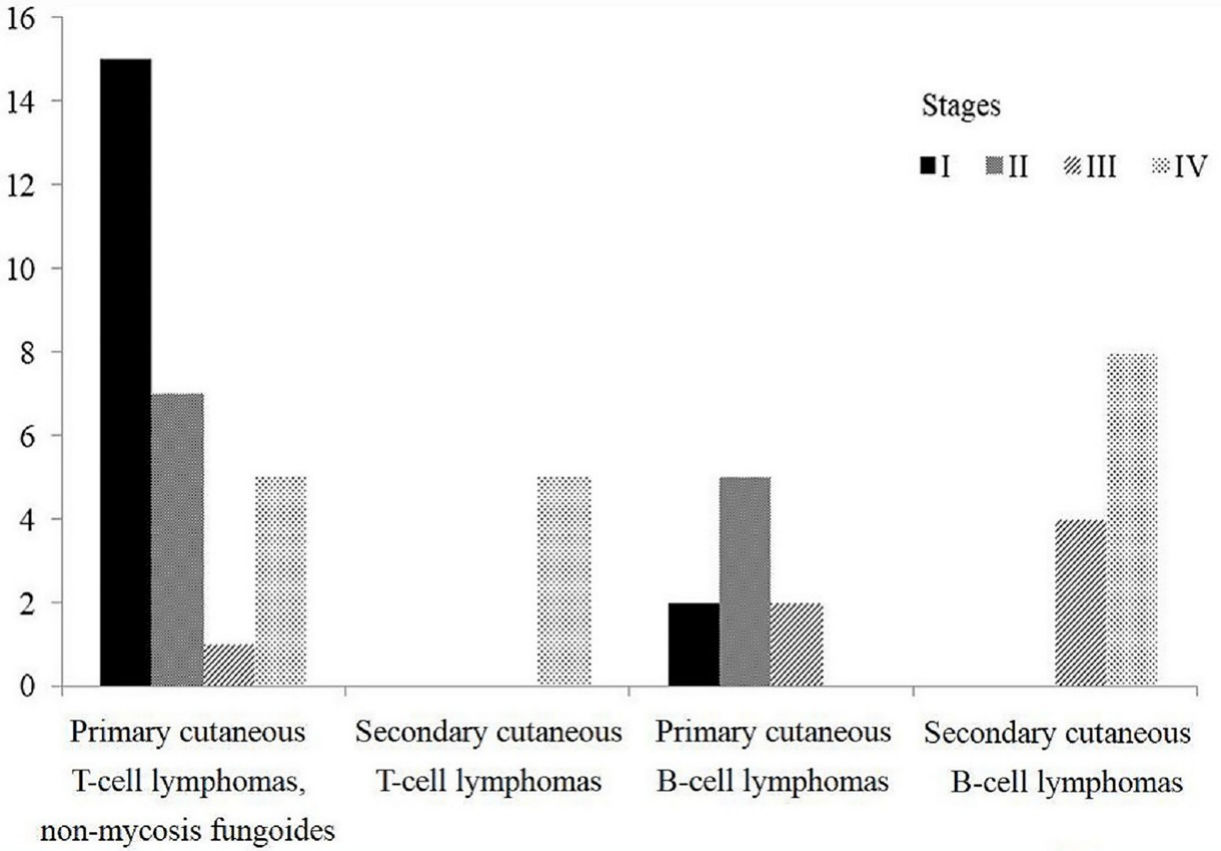
Stages of non-MF/SS CLs in the patients of the present study. The study groups consisted of non-MF/SS primary CTCLs, secondary CTCLs, primary CBCLs, and secondary CBCLs.

Most patients with non-MF/SS primary CTCLs were diagnosed at early stages, specifically, stages I (15 cases) and II (seven cases) (Fig 1). All patients with secondary CTCLs were at stage IV (five cases). The findings were similar in the patients with CBCLs. The patients with primary CBCLs were mainly at stage I (two cases) or II (five cases), while the patients with secondary CBCLs were at advanced stages, specifically, stages III (four cases) and IV (eight cases).

### Survival outcomes

**Fig 2:**
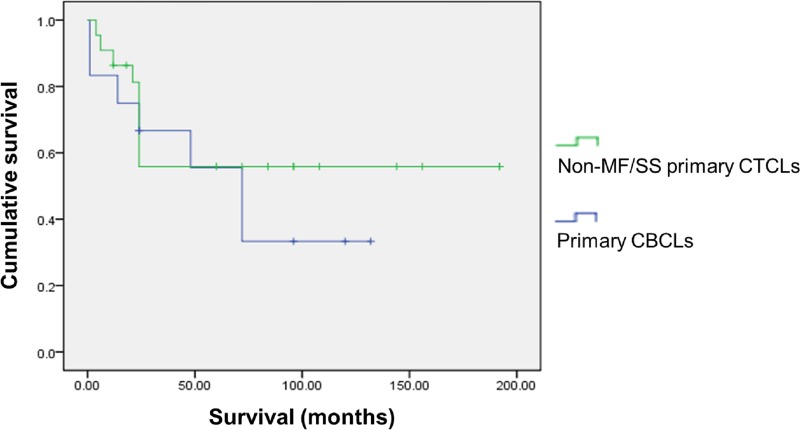
Cumulative survivals in patients with non-MF/SS primary CTCLs and primary CBCLs. The survival outcome of non-MF/SS primary CTCLs was better to that of primary CBCLs.

Overall, the median survival of non-MF/SS CTCLs was 57 months and CBCLs was 41.5 months (Table 2). In non-MF/SS primary CLs, the outcome of CTCLs was better to that of CBCLs (Fig 2), namely 57.5 months in primary CTCLs and 62.5 months in primary CBCLs. In secondary CLs, however, the median survival of CTCLs was better than that of CBCLs, with 43 months and 23 months in secondary CTCLs and secondary CBCLs, respectively. Compared with the median survival of non-MF/SS CTCLs and CBCLs at different stages, there were no obvious differences between CTCLs and CBCLs at early or advanced stages. For early stages, specifically stages I and II, the median survival of non-MF/SS CTCLs was 81 months and CBCLs was 76 months. For advanced stages, specifically stages III and IV, the median survival of non-MF/SS CTCLs was 25.5 months and CBCLs was 26.5 months.

**Table 2:** Median survival time of the patients.

Median survival time (months)	Non-mycosis fungoides natural killer/T-cell neoplasms	Mature B-cell neoplasms
**Overall**	57	41.5
**Primary**	57.5	62.5
**Secondary**	43	23
**Stages I and II**	81	76
**Stages III and IV**	25.5	26.5

### Prognostic factors

**Fig 3:**
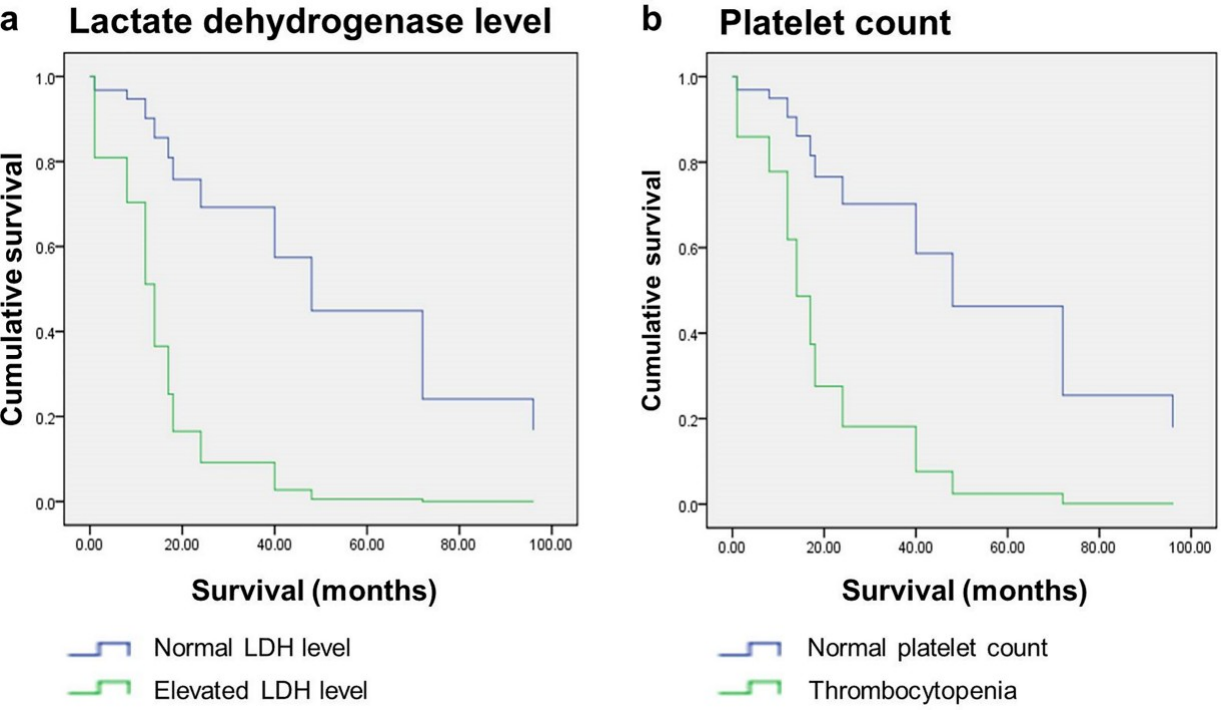
Cumulative survivals of non-MF/SS CLs patients analyzed according to (a) LDH level and (b) platelet count. The survival outcome of non-MF/SS CLs was worse in patients with (a) elevated LDH level and (b) thrombocytopenia.

We assessed the relationship between laboratory features and prognosis in the patients with non-MF/SS CLs. We analyzed survival factors including LDH level, platelet count, albumin level and involved anatomical sites. The survival outcome of non-MF/SS CLs was worse in patients with elevated LDH level (Fig 3A) and thrombocytopenia (Fig 3B). The prognosis of non-MF/SS CTCLs was worse in patients with elevated LDH level (median survival 26.2 months in the group of elevated LDH level and 68.5 months in the group of normal LDH level), thrombocytopenia (median survival 18.5 months in the group of thrombocytopenia and 67.0 months in the group of normal platelet count), multiple anatomical sites of skin involvement (median survival 22.5 months in the group of multiple anatomical sites of skin involvement and 69.8 months in the group of single anatomical sites of skin involvement) and lower albumin level (median survival 17.9 months in the group of lower albumin level and 68.0 months in the group of normal albumin level). While in CBCLs, elevated LDH level (median survival 21.0 months in the group of elevated LDH level and 61.9 months in the group of normal LDH level) and thrombocytopenia (median survival 16.6 months in the group of thrombocytopenia and 59.3 months in the group of normal platelet count) were associated with poorer overall survival. Elevated LDH level, multiple anatomical sites of skin involvement, thrombocytopenia and lower albumin level may be risk factors for poorer prognosis of non-MF/SS CTCLs. Because of a relatively small sample size, statistical analysis could not be performed for each entity of CLs specifically.

## Discussion

### Demographic profiles and comparisons of global regional differences

The present series consisted of 26 patients with MF, 33 patients with non-MF/SS primary CTCLs, five patients with secondary CTCLs, nine patients with primary CBCLs and 12 patients with secondary CBCLs. Though MF and SS were not focused in our study, the two diseases accounted for 38.2% of primary CLs in the present series (Table 3). The ratio was similar to studies from the United States and Germany [4, 12]. In two other studies from Taiwan, MF accounted for 57% of primary CLs reported by Lee et al [10], 48% reported by Chang et al [13], whereas low to 13% reported by Liao et al [8]. There are wide variations in the frequencies of multiple types of CLs including MF from different countries and regions.

**Table 3:** Comparision of the clinical features of present study with previously reported case series of cutaneous lymphomas in the literature.

Clinical features	Taiwan			Japan		Korea		Germany	USA
	Present study	Lee et al [10]	Liao et al [8]	Yasukawa et al [25]	Fujita et al [14]	Lee et al [7]	Park et al [24]	Assaf et al [12]	Bradford et al [4]
**Gender, M:F**	1.18:1	NA	1.3:1	1.8:1	1.25:1	1.2:1	1.34:1	NA	NA
**Non-MF cases (N)**	59	91	49	49	77	46	140	656	2364
**Median age (years)**	60.7	NA	68	62	62	46.2	47.1	NA	NA
**CTCLs (%)**	64	81.4	71	68	80	96.2	79	85	71.3
**CBCLs (%)**	36	18.6	29	32	18	3.8	21	15	28.5
**Primary CL/secondary CL (%)**	80/20	77.1/22.9	55/45	50/50	79.7/20.3	61/39	58.5/41.5	NA	NA
**MF and SS in primary CLs (%)**	38.2	59.3	16.1	41.9	53	69	25	34.3	39.1
**ENKL in primary CLs (%)**	11.8	3.3	16.1	6.4	5	24	16.7	NA	0.3

Abbreviations: CTCL: cutaneous T-cell lymphoma; CBCL: cutaneous B-cell lymphoma; CL: cutaneous lymphoma; MF: mycosis fungoides; SS: Sézary syndrome; ENKL: extranodal NK/T-cell lymphoma; NA: not available

The present series showed a slight male predominance with male/female ratio 1.3 in non-MF/SS CLs. As listed in Table 1, non-MF/SS primary CTCLs, secondary CTCLs and secondary CBCLs all had a male predominance. Only primary CBCLs revealed a female predominance with male/female ratio 0.8. The tendency was similar to data reported in Taiwan [10], Japan [14, 15] and Europe [2]. In our study, the median age of patients with non-MF/SS CLs was 60.5 years old. There was a trend for non-MF/SS primary CTCLs to affect younger males (median age 48 years) and for primary CBCLs to affect elderly females (median age 76 years), compared to 50 years for primary CTCLs and 78 years for primary CBCLs in a Taiwanese study [10], and 64 years for primary CTCLs and 78 years for primary CBCLs in a Japanese study [15]. In a Korean study, analysis of age and sex ratio also revealed patients with CTCLs were significantly younger than those with CBCLs in both primary and secondary CL groups [16].

CTCLs contain many heterogeneous entities. Approximately one-third of CTCLs are designated as pcPTCL-NOS [17]. Gene expression profiling may be helpful to define two major molecular subtypes of pcPTCL-NOS, pcPTCL-GATA3 and pcPTCL-TBX21, which have distinct biological differences and poorer prognosis in pcPTCL-GATA3 [17]. In our study, there were eight cases (13.5% of non-MF/SS CLs and 24.2% of primary CTCLs) classified into pcPTCL-NOS (Table 1). With the progression of immunohistochemical staining and gene expression profiling, the classification of pcPTCL-NOS may be more specific in the future.

### The relative frequencies of CBCLs, ENKL and ATLL with possible roles of viral infections

The proportion of CBCLs in the present series was 36.2%, which was higher than reports of previous studies from other countries. In Korea, the relative frequency of primary CBCLs was significantly higher in the more recent 10-year period from 2004 to 2013 than in the previous 10-year period from 1994 to 2003 [16]. One of the possible explanations is that CBCLs had been reported to be associated with hepatitis B virus (HBV) and hepatitis C virus (HCV) infections. Chronic HBV infection had also been suggested a relationship with the development of NHLs [18, 19]. The odds ratio of HCV infection was significantly higher in B-cell lymphomas in a study from Taiwan [20] and a case-control study from the United Kingdom [21]. The hypothesized pathogenesis of HCV infection in B-cell lymphomas is that HCV-E2 antigen binds to the B-cell surface receptors inducing B-cell clonal expansion [22]. Southern Taiwan was known as a widely endemic area of HBV and HCV infections [23]. The prevalences of hepatitis B surface antigen were 15.1% in Kaohsiung and Pingtung, the two southernmost cities in Taiwan [23], and 17.3% in Taiwan [24]. The prevalence of anti-HCV antibody is 8.6% in the two southernmost cities in Taiwan, and 4.4% in the whole Taiwan [23, 24]. The seropositive rates of HBV and HCV infections in patients with CLs of the present series were higher than that in the general population of previous epidemiologic data, with 50% and 17%, respectively [23, 24]. This finding was consistent with the studies from different countries [18–21].

Our study revealed a higher incidence of ENKL with 13.5% of non-MF/SS CLs and 19% of non-MF/SS primary CLs in Taiwan than that in Western countries [2, 4], comparable to 8.9% of CLs and 16.1% of primary CLs in another Taiwanese study reported by Liao et al [8] (Table 3). The incidences of ENKL were also higher in two studies from Korea (8.5% of CLs and 16.7% of primary CLs) [7, 25], two studies from Japan (3.8% of CLs and 6.4% of primary CLs) [14, 26] and one study from Singapore (5.3% of primary CLs) [27]. While the incidences of ENKL were much lower in Australia (0.003% [2]) and the United States (0.3%) [4]. Epstein-Barr virus (EBV) may play an important role in the development of ENKL, correlated to the expression level of the EBV latent membrane protein 1 [28]. Interestingly, the prevalence of EBV was nearly worldwide without regional differences, but almost 50% of EBV-attributed malignancies and the highest malignancy-related mortality occurred in East Asia [29]. The association between human leukocyte antigen class I genes and EBV-associated malignancies including nasopharyngeal carcinoma and EBV-positive Hodgkin lymphoma had been reported [30]. There are possible factors of EBV genotypes and host genetic factors. Future studies are needed to better understand these patterns in ENKL.

Human T-cell leukemia virus type 1 is endemic in southwestern Japan, sub-Saharan Africa, South America, the Caribbean region, and certain areas in the Middle East and Australia [31]. Human T-cell leukemia virus type 1 causes ATLL in about 5% of the carriers [32]. In the United States, the incidence of ATLL was stable but showed a rising trend in the New York City, which is largely due to non-Hispanic blacks originating from the Caribbean [33]. In our study, the five primary ATLL patients were all Taiwanese. All the patients were carriers of human T-cell leukemia virus type 1.

### Prognostic factors associated with poorer survival outcomes in non-MF/SS CTCLs and CBCLs

An overall prognostic study for non-MF/SS primary CLs revealed that EORTC prognostic group, serum LDH level, B symptoms, and variables related tumor extension such as distribution, maximum diameter, and number of skin lesions were significant factors associated with survival [34]. In the survival analysis of the present series, elevated serum LDH level and thrombocytopenia were significant poor prognostic factors for non-MF/SS CLs. LDH was thought to be an indicator of tumor burden and one of the most important prognostic factors for CLs [35–37]. Thrombocytopenia in lymphomas may due to autoimmune factors, bone marrow involvement or platelet sequestration in the spleen [38, 39]. The condition of thrombocytopenia may get worse and cause morbidity/mortality during treatment courses because of disease progression or toxicity of chemotherapy.

In our study, multiple anatomical sites of skin involvement and low albumin level were associated with poor prognosis in non-MF/SS CTCLs, but not in CBCLs. Multiple anatomical sites of skin involvement is closely associated with advanced stages. More than half of the CBCL patients in present series, namely 12 of 21 cases, were secondary CBCLs. In contrast, there were 33 non-MF/SS primary CTCLs in total 38 cases of non-MF/SS CTCLs in present series and 22 non-MF/SS primary CTCLs were diagnosed at stages I and II (Fig 1). Skin involvement of multiple anatomical sites is an important prognostic factor but may have little effect in secondary CLs due to disseminated nature [40]. Hypoalbuminemia was proposed to be a new prognostic indicator for evaluation of NHLs [41]. In T-cell lymphomas, lower albumin level may reflect cytokine-induced suppression of albumin synthesis and increasing degradation [42].

The median survival of non-MF/SS CTCLs was slightly better than that of CBCLs among overall and secondary cases in the present series. This result may be explained by the age differences between non-MF/SS CTCLs and CBCLs. The median age of non-MF/SS CTCLs (55 years old) was much younger than that of CBCLs (71 years old). Overall, the prognosis of non-MF/SS primary CLs was much better than that of secondary CLs, which tend to had systemic symptoms and complications. In secondary CLs, CTCLs and CBCLs were associated with different prognostic factors [40]. A complete survey is important for CLs to make a definite diagnosis, adequate treatment plan and prediction of survival outcomes.

### Research limitations

Limitations of our study include retrospective nature, referral bias and a relatively small sample size. A large retrospective study based on multicenter analysis or the National Health Insurance Database is needed obtain a comprehensive understanding of the demographic characteristics, relative frequencies, clinical presentations and prognosis of multiple types of CLs in Taiwanese population.

## Conclusions

In conclusions, we reported 59 cases of non-MF/SS CLs diagnosed over a 17-year period from a referral center in southern Taiwan. This study revealed a higher frequency of ENKL in Taiwan compared with Western countries. Thrombocytopenia and elevated LDH level may be associated with poor prognosis for non-MF/SS CLs. Multiple anatomical sites of skin involvement, and lower albumin level may be other unfavorable prognostic factors in non-MF/SS CTCLs. To the best of our knowledge, the present series is the first study focused on non-MF/SS CLs in Taiwan.

## Supporting information

S1 Table Detailed demographic characteristics and survival times the patients. (DOCX)
