# Supplementary material for: Non-mycosis fungoides cutaneous lymphomas in a referral center in Taiwan: A retrospective case series and literature review
Source: PLoS One. 2020 Jan 24;15(1):e0228046. doi: 10.1371/journal.pone.0228046 (PMC6980583; doi:10.1371/journal.pone.0228046)
Supplement: S1 Table — (DOCX) [file pone.0228046.s001.docx]

**S1 Table.** Detailed demographic characteristics and survival times of the patients

| **No.** | **Diagnosis** | **Age** | **Gender** | **Stage** | **F/U time**  **(months)** | | **Skin sites** | | **Treatment** | | |
| --- | --- | --- | --- | --- | --- | --- | --- | --- | --- | --- | --- |
|  | **Primary cutaneous T-cell lymphoma** | | | | | | | | | | |
| 1 | ENKL | 70 | M | 4 | 24 | | Nose, central nervous system | | Chemotherapy | | |
| 2 | ENKL | 80 | F | 2 | 24 | | Face, arms | | Chemotherapy | | |
| 3 | ENKL | 56 | M | 1 | Loss to F/U | | NA | | NA | | |
| 4 | ENKL | 49 | M | 1 | 192 | | Nose | | Chemotherapy, surgery | | |
| 5 | ENKL | 60 | M | 4 | Loss to F/U | | NA | | NA | | |
| 6 | ENKL | 61 | F | 3 | 24 | | Thighs | | Chemotherapy | | |
| 7 | ENKL | 55 | M | 1 | 12 | | Legs | | Chemotherapy, radiotherapy | | |
| 8 | ENKL | 24 | F | 4b | 24 | | Limbs, lips | | NA | | |
| 9 | PTCL-NOS | 37 | M | 2 | 84 | | Thigh, back | | Chemotherapy | | |
| 10 | PTCL-NOS | 57 | M | 2 | 84 | | Face | | Interferon, radiotherapy | | |
| 11 | PTCL-NOS | 75 | M | 2 | Loss to F/U | | Trunk, limbs | | NA | | |
| 12 | PTCL-NOS | 63 | M | 1 | 12 | | Hands, feet | | Chemotherapy | | |
| 13 | PTCL-NOS | 41 | F | 1 | 192 | | NA | | NA | | |
| 14 | PTCL-NOS | 57 | M | 1 | 144 | | Nose | | Spontaneous regression | | |
| 15 | PTCL-NOS | 72 | F | 2 | 156 | | Face, neck, chest | | NA | | |
| 16 | PTCL-NOS | 37 | M | 1 | 18 | | Axillae | | Radiotherapy | | |
| 17 | Primary cutaneous ALCL | 31 | F | 1 | 96 | | Forearms | | NA | | |
| 18 | Primary cutaneous ALCL | 35 | M | 1 | 60 | | Suprapubic | | Surgery | | |
| 19 | Primary cutaneous ALCL | 69 | M | 2 | 6 | | Abdomen | | Chemotherapy | | |
| 20 | Primary cutaneous ALCL | 26 | M | 1 | 72 | | Chest | | Stem cell transplantation | | |
| 21 | Primary cutaneous ALCL | 12 | F | 1 | Loss to F/U | | NA | | NA | | |
| 22 | Primary cutaneous ALCL | 54 | M | 1 | 24 | | Hand | | NA | | |
| 23 | Primary cutaneous ALCL | 14 | M | 1 | 12 | | NA | | Surgery | | |
| 24 | Primary cutaneous ALCL | 31 | F | 1 | Loss to F/U | | Face | | NA | | |
| 25 | ATLL | 71 | F | 4 | 6 | | Feet | | Chemotherapy | | |
| 26 | ATLL | 66 | M | 4 | 1.3 | | Feet | | Chemotherapy | | |
| 27 | ATLL | 74 | M | 4 | 4.5 | | Trunk, legs | | Chemotherapy | | |
| 28 | ATLL | 61 | F | 4 | 10 | | Face, neck | | Chemotherapy | | |
| 29 | ATLL | 61 | F | 4 | 1.7 | | Generalized | | Chemotherapy | | |
| 30 | SPTCL | 35 | F | 1 | 108 | | Nape, forearms | | Chemotherapy | | |
| 31 | SPTCL | 37 | F | 4b | 4 | | Perianal area | | Chemotherapy | | |
| 32 | SPTCL | 51 | F | 2 | 96 | | Thighs | | Chemotherapy | | |
| 33 | SPTCL | 58 | F | 4b | 21 | | Chest, abdomen | | Chemotherapy | | |
|  | **Secondary cutaneous T-cell lymphoma** | | | | | | | | | | |
| 34 | Angioimmunoblastic T-cell lymphoma | 46 | M | 4b | 48 | | Chest, back | | Chemotherapy | | |
| 35 | Angioimmunoblastic T-cell lymphoma | 64 | F | 4 | 96 | | Face | | Chemotherapy | | |
| 36 | Angioimmunoblastic T-cell lymphoma | 48 | M | 4 | 48 | | Legs | | Chemotherapy | | |
| 37 | Angioimmunoblastic T-cell lymphoma | 71 | M | 4b | 24 | | Face, trunk | | Chemotherapy | | |
| 38 | Systemic ALCL | 26 | F | 4 | 1 | | Thighs | | Chemotherapy | | |
|  | **Primary cutaneous B-cell lymphoma** | | | | | | | | |  |  |
| 39 | Primary cutaneous DLBCL | 59 | F | 2a | 24 | | Chest | | Chemotherapy | |  |
| 40 | Primary cutaneous DLBCL | 76 | M | 1 | 132 | | Arms, legs | | Chemotherapy | |  |
| 41 | Primary cutaneous DLBCL | 47 | M | 1 | 120 | | Cheeks | | Surgery | |  |
| 42 | Primary cutaneous DLBCL | 76 | F | 2 | 96 | | Lower abdomen | | Chemotherapy | |  |
| 43 | Primary cutaneous DLBCL | 81 | F | 2 | 72 | | Face, neck, trunk | | Chemotherapy | |  |
| 44 | Primary cutaneous DLBCL | 86 | M | 2 | 14 | | Legs | | Chemotherapy | |  |
| 45 | Extranodal marginal lymphoma of mucosa associated lymphoid tissue (MALT lymphoma) | 52 | M | 2 | 12 | | Arms, abdomen, back | | Chemotherapy | |  |
| 46 | Primary cutaneous follicle center lymphoma | 76 | F | 3 | 72 | | Chest | | Chemotherpy | |  |
| 47 | Intravascular large B-cell lymphoma | 71 | F | 3b | 24 | | Legs | | Chemotherapy | |  |
|  | **Secondary cutaneous B-cell lymphoma** | | | | | | | | | | |
| 48 | DLBCL | 68 | F | 4 | 96 | Neck lymph nodes, arms | | Chemotherapy | | | |
| 49 | DLBCL | 70 | M | 3b | 48 | Abdomen, inguinal area | | Chemotherapy | | | |
| 50 | DLBCL | 40 | F | 4 b | 9 | Face, trunk | | Chemotherapy | | | |
| 51 | DLBCL | 35 | F | 4 | 18 | Neck, axilla | | Chemotherapy, radiotherapy | | | |
| 52 | DLBCL | 55 | M | 4b | 17 | Arms | | Chemotherapy | | | |
| 53 | DLBCL | 66 | F | 4a | 40 | Forearms | | Chemotherapy | | | |
| 54 | DLBCL | 66 | M | 4a | 8 | Face, neck | | Chemotherapy | | | |
| 55 | Mantle cell lymphoma | 71 | M | 3 | 24 | Limbs | | Chemotherapy | | | |
| 56 | Mantle cell lymphoma | 71 | M | 3 | 1 | Limbs | | Chemotherapy | | | |
| 57 | Mantle cell lymphoma | 60 | M | 4 | 1 | Legs | | Chemotherapy | | | |
| 58 | MALT lymphoma | 60 | M | 4 | 1 | Legs | | Chemotherapy | | | |
| 59 | Follicular center lymphoma | 65 | F | 4 | 12 | Axillae | | Chemotherapy | | | |

Abbreviations: F/U: follow-up; ENKL: extranodal NK/T-cell lymphoma; PTCL-NOS: peripheral T-cell lymphoma, not otherwise specified; ALCL: anaplastic large-cell lymphoma; ATLL: adult T-cell leukemia/lymphoma; SPTCL: Subcutaneous panniculitis-like T-cell lymphoma; DLBCL: diffuse large B cell lymphoma; NA: not available
